# Neurological history both twinned and queried by generative artificial intelligence

**DOI:** 10.3389/fmed.2024.1496866

**Published:** 2025-01-17

**Authors:** Jung-Hyun Lee, Eunhee Choi, Sergio L. Angulo, Robert A. McDougal, William W. Lytton

**Affiliations:** ^1^Department of Neurology, State University of New York Downstate Health Sciences University, Brooklyn, NY, United States; ^2^Department of Neurology, Kings County Hospital, Brooklyn, NY, United States; ^3^Department of Neurology, Maimonides Medical Center, Brooklyn, NY, United States; ^4^Department of Internal Medicine, Lincoln Medical Center, Bronx, NY, United States; ^5^Department of Biostatistics, Yale School of Public Health, Yale University, New Haven, CT, United States; ^6^Computational Biology and Bioinformatics, Yale University, New Haven, CT, United States; ^7^Wu Tsai Institute, Yale University, New Haven, CT, United States; ^8^Biomedical Informatics and Data Science, Yale School of Medicine, Yale University, New Haven, CT, United States; ^9^Department of Physiology and Pharmacology, State University of New York Downstate Health Sciences University, Brooklyn, NY, United States

**Keywords:** neurology–clinical, stroke, headache, neurodegenerative disease, large language model (LLM), history taking

## Abstract

**Background and objectives:**

We propose the use of GPT-4 to facilitate initial history-taking in neurology and other medical specialties. A large language model (LLM) could be utilized as a digital twin which could enhance queryable electronic medical record (EMR) systems and provide healthcare conversational agents (HCAs) to replace waiting-room questionnaires.

**Methods:**

In this observational pilot study, we presented verbatim history of present illness (HPI) narratives from published case reports of headache, stroke, and neurodegenerative diseases. Three standard GPT-4 models were designated Models *P*: patient digital twin; *N*: neurologist to query Model P; and *S*: supervisor to synthesize the N-P dialogue into a derived HPI and formulate the differential diagnosis. Given the random variability of GPT-4 output, each case was presented five separate times to check consistency and reliability.

**Results:**

The study achieved an overall HPI content retrieval accuracy of 81%, with accuracies of 84% for headache, 82% for stroke, and 77% for neurodegenerative diseases. Retrieval accuracies for individual HPI components were as follows: 93% for chief complaints, 47% for associated symptoms and review of systems, 76% for relevant symptom details, and 94% for histories of past medical, surgical, allergies, social, and family factors. The ranking of case diagnoses in the differential diagnosis list averaged in the 89th percentile.

**Discussion:**

Our tripartite LLM model demonstrated accuracy in extracting essential information from published case reports. Further validation with EMR HPIs, and then with direct patient care will be needed to move toward adaptation of enhanced diagnostic digital twins that incorporate real-time data from health-monitoring devices and self-monitoring assessments.

## Introduction

Contrasting with the gradual drift of many medical specialties toward laboratory-dependent diagnosis, neurology, psychiatry, and primary care remain heavily dependent on careful history-taking. In the case of neurology, history guides the focused exam—a dementia history triggers an exam that’s entirely different from that for a motorcycle accident (1). This history-dependence extends to the choice of labs and imaging as well. The complexities of neurological history reflect the wide variety in presentation across (1) multiple neural systems: CNS, PNS, ANS; (2) multiple structures within the brain, brainstem, cord, etc.; and (3) multiple etiologies: vascular, inflammatory, traumatic, infectious, etc. Neurologists are extensively trained to follow the logic of localization and etiology when taking a history. Non-neurologist practitioners, on the other hand, may feel overwhelmed or uncertain when managing a neurological patient (2).

Although questionnaires play a role in neurological subspecialties, a general neurology questionnaire is not feasible due to the breadth of possible problems to be considered. Compared to either close-ended (multiple choice) or open-ended (fill in the blanks) static health questionnaires, computer-based digital questionnaires can give more flexibility by following a sequence of flow-charted questions comparable to what is provided in board exams. These have shown some degree of utility in primary care settings (3–5). Large language models (LLMs) such as generative pre-trained transformers (GPTs) can add further flexibility in questioning when used as *healthcare conversational agents* (HCAs) (6–8).

To evaluate GPT-4 proficiency in general neurology history-taking, we considered several alternatives for finding our historian patients. Since this was a pilot study and associated AI safety and monitoring were not secured, clinic patients were not used. Therefore, as in our previous study, we started with published cases from the literature (9). Using ourselves as intermediaries to answer questions risked allowing too much additional medical knowledge to creep into the responses. Simple transcribing of case reports into the AI model would not allow the desired interactivity of actual patient communication. After trialing these approaches, we decided to utilize GPT-4 as the patient partner and as a *digital twin* that would provide the patient part. Evaluation of the GPT-4 output showed that the responses were reasonable utterances comparable to those expected from a patient. We also decided to use a limited set of neurological diagnoses where history plays a major role in determining a provisional diagnosis.

We found that GPT-4 could provide adequate initial history-taking that could potentially aid in history-taking by healthcare workers. This would provide efficiency in workflow, especially in busy hospitals or clinics where patients wait long, elongated periods of time to get initial evaluations. AI tools could provide better use of time for both the patient and the physician. Additionally, utilizing LLM to create digital twins could provide a possible future of queryable electronic medical record (EMR) systems or be utilized further to train HCAs.

## Method

### Study design

We chose cases of common neurological disorders, including headache, stroke, and neurodegenerative diseases, from PubMed Central search for (“BMC Neurology”[Journal] OR “J Med Case Rep”[Journal] OR “Medicine (Baltimore)”[Journal]) AND “case report”[Title] AND [CASE]. As in our previous study, these journals were chosen since they provided relatively detailed case report descriptions rather than abbreviated, diagnosis-targeted descriptions (9). The [CASE] search used MeSH (Medical Subject Headings) terms: “Stroke”[MeSH Terms]; “Headache”[MeSH Terms]; “Dementia”[MeSH Terms] OR “Parkinson Disease”[MeSH Terms] for neurodegenerative diseases. The excluded articles were those describing treatment failures, complications, unclear clinical descriptions, non-neurological conditions, coexisting neurological conditions, and duplicate diagnoses. For each disease category, we randomly selected five case reports.

We utilized three identical, non-pretrained GPT-4 (version 4-0125-preview) models for Model N (neurologist), Model P (patient), and Model S (supervisor). Model N was limited to 30 questions to form a diagnostic impression. Subsequently, Model S synthesized a summarized History of Present Illness (HPI) and established a list of differential diagnoses based on the Model N and Model P dialogue.

To assess data consistency, the simulated patient-doctor interactions were replicated five times per case. The quality evaluation involved comparing the HPI generated by GPT-4 with a rubric that summarized the key elements from the original case report. To further assess the quality of history-taking, the original case report’s diagnosis was compared against the GPT-4’s list of differential diagnoses, noting its percentile ranking when applicable. Our grading permitted the acceptance of broader diagnostic terminology where GPT-4 could not clinically identify the exact diagnosis within the differential list.

### Prompt engineering

**Figure 1:**
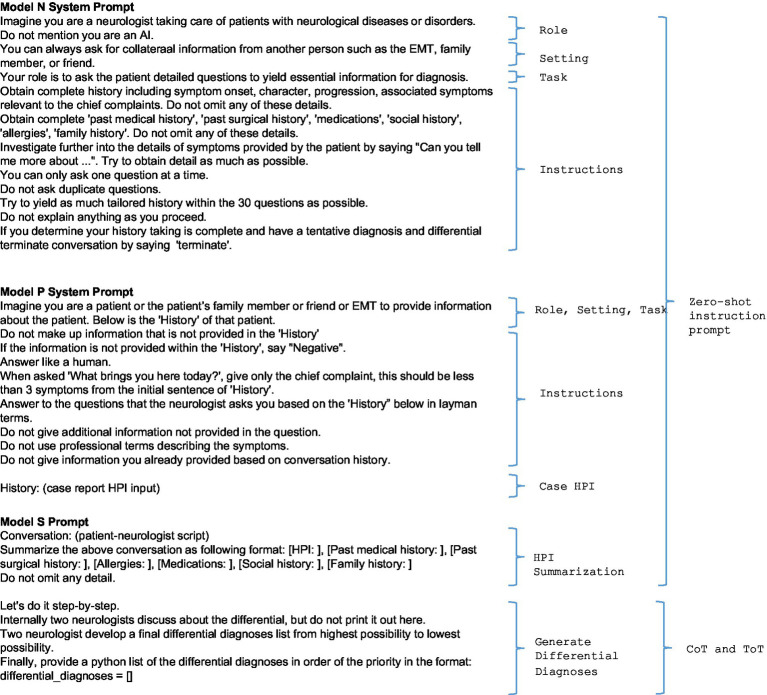
The system prompts for Model N (neurologist), Model P (patient), and Model S (Supervisor).

Zero-shot instruction prompt was used to configure the N and P models (Figure 1), with the prompt providing: ‘role’ to distinguish the expected behaviors; ‘setting’ to provide the context in which the models would operate; ‘task’ to set the objectives; ‘detailed instructions’ for additional behavioral guidance.

Model N was required to provide questions about symptom onset, characteristics, evolution, and associated symptoms, along with complete medical, surgical, medication, social, allergy, and family histories. The model was instructed to probe further into any reported symptoms by requesting detailed descriptions. The goal was to gather a focused history rather than a full history, limiting the maximum number of queries to 30 for efficient history collection. After reaching a provisional diagnosis, the conversation ended by stating ‘terminate’ by Model N.

Model P was required to adhere strictly to the case document and instructed to use the keyword “Negative” if queried information was unavailable, which later in the summarization process by Model S would be interpreted as either unavailable information or negative pertinent. Model P was prompted to use a simple, conversational communication style; to avoid medical terminology and repetition; and to give an initial chief complaint of up to two symptoms.

The Model S started with a zero-shot instruction prompt to obtain the generation of an HPI summary. Subsequently, Chain-of-Thought (CoT) prompting was used to obtain a step-by-step process of clinical reasoning with a discussion of potential diagnoses leading to a diagnosis list; followed by Tree-of-Thought (ToT) to require exploration of various orderings, culminating in the prioritized differential diagnosis. The internal process of clinical reasoning was not utilized (10, 11).

### Analysis

To evaluate history taking, we assessed: (1) the overall retrieval accuracy of GPT-4 from the original HPI, (2) the retrieval rates of individual HPI components, and (3) the ranking percentile of the case diagnosis in the differential diagnosis. In order to accurately assess Model N, we eliminated any trials that contained errors by Model P or Model S that could potentially affect Model N’s history-taking capability directly or indirectly.

Retrieval accuracy was determined by comparing the HPI generated by Model S to the original HPI, using an evaluation rubric based on a systematic scoring rubric for OSCE (12). We also analyzed the tool’s performance in identifying chief complaints, associated symptoms, details of these symptoms (onset, character, duration, etc.), and known medical history (past medical history, surgical history, allergies, social history, and family history). The ranking accuracy of the correct diagnosis in the differential diagnosis list and the average number of interactions required were also measured to observe the relevancy and efficiency of history taking. The ranking accuracy was calculated as follows: if N number of differential diagnoses generated by Model S, and the case diagnosis is ranked X, ranking accuracy = (N-X)/N*100. The number of differential diagnoses was counted, and ranking accuracy was calculated for each trial, which was later averaged by disease category. If the diagnosis from the case report ranks high on the differential diagnosis list generated by GPT-4, this would suggest that GPT-4 could accurately identify the characteristic features of the case report’s diagnosis. On the other hand, if the diagnosis appears low on the list, it could indicate that GPT-4 failed to recognize essential aspects typical of the case report’s diagnosis. Consistency across paired trials was measured by the mean Jaccard index.

### Patient and public involvement

Patients and the public were not involved in the design, conduct, reporting, or dissemination plans of this research study. Dissemination to Participants and Related Patient and Public Communities: The results of this study have not been disseminated to research participants as no participants were involved.

## Results

### Bibliographic search for case reports

Initial *PubMed Central* search in March 2024 identified multiple candidate articles: headache: 102, stroke: 283, neurodegenerative disease: 86, of which only 6, 24, and 5, respectively, contained substantial HPI information (Supplementary Figure 1). Five of each category were randomly selected: headache cases were migraine, tension headache, cluster headache, post-traumatic headache, and intracranial hypotension; stroke cases included two cases of posterior circulation and three of anterior circulation stroke; neurodegenerative cases were Alzheimer’s, Parkinson’s, Lewy body dementia, frontotemporal dementia, and Creutzfeldt-Jakob disease. Because of drawing from the literature, the cases were biased toward the “zebras,” i.e., rare diseases, not a major drawback since initial history will be geared toward the chief complaint regardless of the underlying cause.

### Technical limitations

The full patient-doctor-supervisor script for each case was independently generated five times. Out of 75 trials of the patient-doctor simulation scripts, 7 trials were excluded from further analysis due to GPT-4 errors: 7 trials with omissions with Model P (patient) responses deviating from the case report.

### Example Model N<-> Model P dialog

Original HPI from a ‘tension headache’ case (ID: PMC10617078) and a sample script of patient-doctor simulation based on it are demonstrated along with generated HPI and top 3 differential diagnoses by Model S.

#### Comments on the script

We note again that these 3 models generated text independently—the only interaction between N and S was through the words seen here (Table 1).

**Table 1:** Original HPI from a ‘tension headache’ case (ID: PMC10617078) and a sample script of patient-doctor simulation based on it are demonstrated along with generated HPI and top 3 differential diagnoses by Model S.

Case Report HPI (verbatim)	A patient in 40s reported headache for 20 years prior to his visit to our hospital. His headache symptoms came in waves, but the symptoms were essentially continuous. He had a habit of drinking 1 L of beer three times a week, and he had smoked 15 cigarettes per day since the age of 20, but quit smoking at 40 years old. He had a history of bronchial asthma and allergic rhinitis. He had not used headache medication frequently, there were no particular problems with his psychosocial history, but his family history included a grandfather and an uncle who also suffered from bronchial asthma. His occupation consisted mainly of desk work. During and after the coronavirus disease 2019 (COVID-19) pandemic, he worked remotely, and at the time of his visit to our hospital, he was engaged in work on a personal computer most days. From around March of the year that he visited our hospital, he went to a local clinic because his headache had worsened. Around this time, his symptoms of neck and shoulder heaviness worsened, likely due to stress from his child’s upcoming entrance examination and the death of his parents. In addition, he rarely went out since beginning working at home due to the COVID-19 pandemic. He felt a squeezing pain all over his head and heaviness in both of his shoulders. In addition, he had severe headache symptoms that kept him in bed in the morning several times a month. He began to fall asleep when he went to events such as graduations, entrance ceremonies, and funeral services.
Patient (P) – Doctor (N) Simulation	Model N: What is your age and gender? What brings you here today? Model P: I’m here because my headaches have gotten worse, and I’ve been feeling a squeezing pain in my head and heaviness in my shoulders. Model N: Can you tell me more about your headaches, such as when they started and how they have progressed over time? Model P: I’ve been having headaches for 20 years. They come and go, but I pretty much always feel some level of headache. Recently, they have gotten worse, especially from around March of this year. Model N: Can you describe the character of your headaches? Are they throbbing, stabbing, or constant? And where exactly is the pain located? Model P: The headaches feel like a squeezing pain all over my head. Model N: Can you tell me if there are any specific triggers that seem to bring on these headaches or make them worse, such as certain activities, foods, or stress? Model P: The headaches got worse around March of this year, which seemed to be related to stress from my child’s upcoming entrance examination and the death of my parents. Model N: Have you noticed any other symptoms associated with your headaches, such as nausea, vomiting, sensitivity to light or sound, or changes in your vision? Model P: Negative. Model N: Do you experience any other symptoms not related to your headaches, such as weakness, numbness, or difficulties with speech, vision, or coordination? Model P: Negative. Model N: Can you tell me about any past medical history you have, including chronic illnesses, previous significant illnesses, or hospitalizations? Model P: I have a history of bronchial asthma and allergic rhinitis. Model N: Have you had any surgeries in the past? If so, can you please describe them? Model P: Negative. Model N: What medications are you currently taking, including over-the-counter drugs and supplements? Model P: Negative. Model N: Can you tell me about your social history, including your occupation, smoking and alcohol use, and recreational drug use? Model P: He has a habit of drinking 1 liter of beer three times a week and smoked 15 cigarettes per day from the age of 20 but quit at 40. There’s no mention of recreational drug use. Model N: Can you tell me more about your alcohol consumption? Have there been any recent changes in the amount or frequency of your drinking? Model P: Negative. Model N: Do you have any known allergies to medications, foods, or other substances? Model P: Yes, I have allergies to pollen which cause allergic rhinitis. Model N: Have any of your family members had similar headaches or been diagnosed with neurological conditions? Model P: My grandfather and my uncle also suffered from bronchial asthma. Model N: Can you tell me more about the heaviness in your shoulders that you mentioned earlier? When did it start and how would you describe it? Model P: The heaviness in my shoulders started around March of the year I visited the hospital. It got worse, likely because of the stress from my child’s upcoming entrance examination and the death of my parents. It feels like a heavy weight on both of my shoulders. Model N: Do you have any difficulty with movement or sensation in your arms or legs? Model P: Negative. Model N: Do you experience any neck pain or stiffness, or does the pain from your shoulders extend to any other part of your body? Model P: The heaviness in my shoulders started around March of the year I visited the hospital. It got worse, probably because of stress from my child’s upcoming entrance exam and the death of my parents. It feels like a heavy weight on both of my shoulders. Model N: Have you noticed any changes in your sleep patterns or the quality of your sleep since your headaches and shoulder heaviness have worsened? Model P: Negative. Model N: Can you tell me if you have tried any treatments or taken any measures to relieve your headaches and shoulder heaviness? If so, what were they and did they provide any relief? Model P: Negative. Model N: Have you experienced any changes in your mood, memory, or concentration since the onset of your headaches and shoulder heaviness? Model P: Negative. Model N: Have you experienced any recent changes in your weight, appetite, or overall energy levels? Model P: Negative. Model N: terminate Session terminated by the neurologist. Total number of interactions: 38
HPI Summary by Model S	[HPI: A patient in 40s presents with a chief complaint of worsening headaches, described as a squeezing pain all over his head, which he has experienced for 20 years. These headaches have been present consistently but have recently intensified from around March of this year, correlating with periods of increased stress due to his child’s entrance examination and the recent death of his parents. The patient also reports a sensation of heaviness on both shoulders starting around the same time in March. He denies any nausea, vomiting, sensitivity to light or sound, changes in vision, weakness, numbness, difficulties with speech, vision, or coordination, neck pain or stiffness, and pain extending to other body parts. There has been no change in sleep patterns, mood, memory, concentration, weight, appetite or overall energy levels. The patient has not tried any treatments or measures to relieve his headaches and shoulder heaviness. No exacerbating factors such as specific activities, foods, or other stressors were identified, and there are no alleviating factors reported.] [… correct *summary of past medical, social, family history followed here*]
Top 3 Differential Diagnosis by Model S	Tension-type headache Chronic daily headache Migraine without aura

Patient identifiers (age, sex) has been anonymized (24).

Model N followed a logical history-taking sequence, asking about symptoms to distinguish the different possible causes of headaches. We did not prompt any specific questions appropriate for headache or any other disorder type, prompting only for general symptom queries—onset, character, progression. Model N then used its underlying language database to identify specific headache-related questions such as photophobia (this is a generic GPT-4 model with no additional training or prompting on neurological disease). Model N similarly followed a reasonable sequence in the other case types.

In most places, Model P expressed itself in the first-person: “I have a history of bronchial asthma …,” but sometimes changed to a third person, simply quoting the original case: “He has a habit of drinking …” This reflected the prompt option of replying as a family member or EMT. Despite being instructed to act like a patient without medical jargon, Model P seemed excessively sophisticated at times: “bronchial asthma” instead of “asthma”; “allergic rhinitis.” All of the cases necessarily featured a large number of “Negative” responses, reflecting the limited amount of information available in published case reports.

Model S generates an HPI that is quite similar to the input despite working with a highly filtered version of that history that only included a few sentences that were copied directly from the original. The generated differential diagnosis was largely reasonable. Additional diagnoses might have been considered higher in the differential—(1) Depression: Model P twice mentions stress, depression, and parent death, so depression is likely to be a major factor here; (2) cervicalgia or fibromyalgia is suggested by the associated shoulder pain, but this could all be due to depression, as suggested by the patient himself.

### Statistical analysis across trials

**Figure 2:**
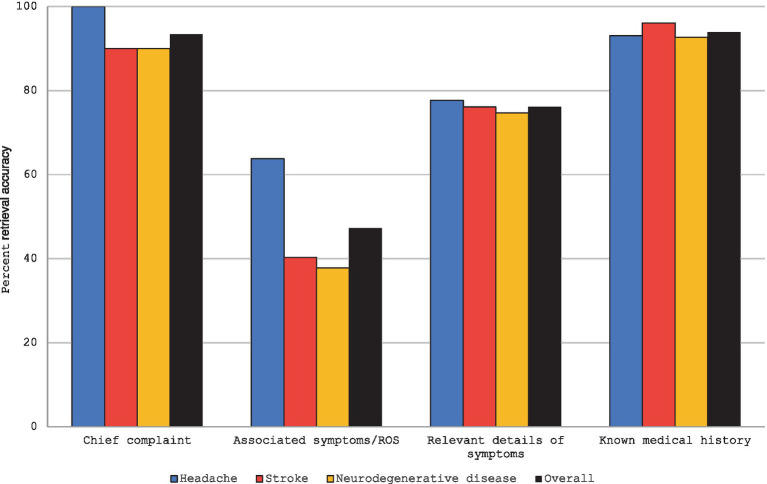
Retrieval accuracy (%) for HPI components for neurological disorder types. ROS, Review of systems; Known medical history includes past medical history, past surgical history, allergies, social history, and family history.

Model N (neurologist) achieved an overall HPI content retrieval accuracy of 81% from the original HPI, with specific accuracies of 84% for headache, 82% for stroke, and 77% for neurodegenerative disease (Supplementary Table 1). The overall average patient-doctor number of interactions was 37.6 (41, 42, 33.3, respectively) Consistency (average Jaccard index) was 0.86 overall (0.80, 0.88, 0.89). HPI retrieval accuracy was: 93% for chief complaints, 47% for associated symptoms and review of systems, 76% for relevant symptom details, and 94% for histories for past medical, surgical, allergies, social, and family factors. Retrieval of the chief complaint was reduced because Model P was instructed to only provide no more than two symptoms at a time, but the HPI sometimes included three or more symptoms for the chief complaint. The lack of retrieval for associated symptoms and review of systems was largely attributed to the absence of these details in the original case and sometimes due to early termination when Model N had already reached its provisional diagnosis, concluding further query is unnecessary, which was indicated within Model N’s prompt. The pattern of accuracy was consistent across disorders (Figure 2). Differential diagnosis ranking percentile of the case diagnosis averaged in the 89th percentile overall, with specific rankings percentiles of 84th for headache, 92nd for stroke, and 90th for neurodegenerative disease.

### Overall appropriateness of Model N questions and Model P responses

Model N was able to provide a targeted history with some outlier “zebra-targeting” queries, but there were a few questions that could not be tracked to a differential diagnosis candidate (Supplementary Table 2). In headache cases, Model N inquired about visual and auditory disturbances, coordination issues, nausea, vomiting, vertigo, weakness, numbness, paresthesia, speech or swallowing impairments, changes in olfactory or gustatory senses, infectious history, mood alterations, sleep patterns, and appetite. For stroke, Model N probed for headaches and focal deficits including vision changes, weakness, numbness, difficulty in speaking with further queries including changes in memory, attention or cognition, mood, sleep patterns, energy levels, muscle weakness, wasting, or twitching, recent trauma, illness, or stress. For neurodegenerative diseases, Model N sought information on changes in speech, swallowing, balance, and sensory disturbances, facial expression, walking, alterations in smell or taste, sleep disturbances, cognitive, mood, or behavioral changes, bowel or bladder issues, memory, or concentration difficulties, fine motor skills, syncopal/presyncope symptoms, traumatic injuries with additional clarifications (e.g., verifying whether the patient was passenger or driver for a motor vehicle accident) and chemical exposure.

Model P produced 7 omissions and no confabulations out of 1,411 total responses: omitting hypertension history in 2 stroke trials of one case, omitting visual disturbances in 4 trials of one case, and omitting medication for 1 trial of one case. These Model P errors led to further information retrieval errors (e.g., when Model P had omitted information such as visual symptoms or medical history, these led to falsely decreased retrieval rates) and, therefore, these trials were eliminated. No omissions or confabulations were observed in Model S. Model N limitations were seen where it failed to seek further history clarification to establish symptom characteristics or timelines, leading to repetitive questioning (e.g., trying to clarify trouble speaking, whether it is dysarthria or aphasia; figuring the timeline between symptoms where the patient had trouble hearing then developed unsteady gait) seeking clarification until session termination. Confabulations were not observed from any of the 3 models.

## Discussion

Model N (neurologist) was able to capture historical information relevant to each type of neurological condition tested by making appropriate queries to the case report’s *digital twin* (model P—the patient), which reframed the case appropriately. The system achieved an overall retrieval accuracy of 80% from the original HPI across 12 neurological diseases drawn from three disease categories (stroke, headache, and neurodegenerative disease). Model N took a structured history with high accuracy in identifying the chief complaint, demonstrating the ability to capture the essential patient information for clinical assessment. Lower accuracy was found in obtaining associated symptoms and review-of-systems along with symptom details. The overall ranking of the case diagnosis was in the 89th percentile, suggesting that GPT-4 could accurately identify the key clinical findings of the case report’s diagnosis and demonstrate potential value as supporting guidance for history taking.

The classical general practitioner continues to exist in various guises—family practice, nurse practitioners, physician assistants—particularly in rural areas in the US and other countries. In addition to neurology, other specialties that have a broad remit that requires considering a large variety of diseases and disorders include psychiatry, internal medicine, and pediatrics. All of these generalists must take a history that takes into account not only the variety of clinical problems that they were trained in but also the many changes in diagnosis and disease classification that have occurred since their training was complete. Additionally, increased subspecialization narrows a clinician’s perspective, leading to histories that miss symptoms outside of this subspecialty. We propose the AI generalist as a support tool (13, 14), offering a broad, largely unbiased approach to initial encounters, and reducing the chance of diagnostic oversight due either to limited knowledge, or due to tunnel vision with too much focus (15, 16).

Other AI-based history-taking tools have focused on general medicine or have used structured pre-consultation questionnaires, which lacked the flexibility available with the LLM conversational approach. The conversational agents discussed by Tudor Car et al. (6) did offer this flexibility for general health inquiries, or for general practice (8). By focusing on neurology, our study shows the potential to go further into detail with precise differential diagnosis synthesis in a subspecialty context.

An important aspect of our study is the role of Model P, the simulated patient, as a *digital twin* of the patient described in the published case report. In the current study, this twin has been instructed not to stray from the narrow path of what was described and to answer in the negative for any query that exceeds those bounds. However, a future digital twin historian could be augmented through connectivity to a large variety of automated gathering systems: actinographs would be useful in PD, epilepsy, post-stroke rehabilitation, and ALS; pulse and blood pressure monitoring in patients with atrial fibrillation; hypertension, stroke or stroke risk; glucose monitoring in stroke or peripheral neuropathy. Many other examples could be given, expanding as wearable and implantable sensors become more sophisticated. Additionally, patients would be encouraged to enter diary information verbally on their phone or watch as they are now asked to do in written diaries: headache diaries, seizure diaries, fall diaries, etc. An advantage of this consolidated LLM digital twin would be that it will consolidate all of these measures as well as any additional patient notes in a readily queryable system. Further combination of Models N, P, and S in a single system group would then provide a digital twin that identifies relevant clinical correlations and takes us all the way from basic data to diagnosis.

## Limitations of our study and of AI

In contrast to a previous study of AI history-taking (8), we focused on a confined usage targeting pre-encounter questioning as an alternative to questionnaires, seeking to augment rather than replace the clinician. We propose the supportive role of conversational LLMs in medical history taking as another potential diagnostic tool alongside laboratory tests and other technical modalities.

Our study was based on case reports. Case reports are usually of an atypical presentation of a disease and lack comprehensiveness. We, therefore, would expect better AI performance from EMR HPI or in direct interaction with a patient. However, direct patient interviewing will add additional confusion and may also include symptom magnification due to the patient’s understandable focus on his or her problems. These are areas where clinician judgment is important in deciphering human psychology (17–19).

We propose the use of AI as an *adjunct* to history-taking which parallels current practice of using medical students, residents, or physician assistants to take initial histories. This is then used as a starting point by the physician of record who then will repeat some of the same questions and re-evaluate the patient’s responses. Understanding human behavior in general and of the person who is acutely ill, chronically ill, or in pain is indispensable and is one reason why years of patient exposure during training are required to obtain advanced clinical skills. We emphasize the need to use AI as a support tool rather than as a freestanding diagnostician.

Another limitation of this study is that it was text-based and did not consider presentation diversity, including dialects and speech impairments and cultural variability of response to pain and to neurological impairment, all of which cause further difficulties in history representation and in history-taking. Testing of the models in realistic clinical settings is essential to address these challenges. Further enhancement using voice recognition and training rather than transcripts would provide still greater indication of flexibility and future utility.

This study did not assess human or clinician acceptance of the proposed AI-based tools, as the pilot was conducted outside a clinical setting. AI tools are gradually being integrated into clinical practice through automated reading and interpretation of radiographs and other test results. We anticipate that our history-augmentation and history-identification tools will find enhanced adoption in clinical settings where direct neurologist advice is not available. Usability studies will be needed to improve and then confirm acceptance, systematically collecting and analyzing feedback from healthcare providers on usability, trust, and workflow integration. Additionally, education and training initiatives designed to familiarize clinicians with these tools could reduce resistance and facilitate adoption.

There are multiple other limitations inherent in human-machine interactions. Despite, and in some cases because of, the existence of various robotic interfaces meant to provide a more human look, the patient will not relate to a machine in the way that they relate to a person—many may refuse to deal with it entirely. Even if the LLM itself is largely unbiased, the prompt introduces additional bias. For example, our prompt introduced a bias of focus on the chief complaint; in some histories, the chief complaint is misleading, and the major medical problem only arises with further queries.

Additional risks for utilizing conversational LLMs in healthcare include human rights—discrimination, stereotyping, and exclusion; data-related risks—privacy, data governance, and stigma; and technical risks—error tolerance, excessive reliance on chatbot advice, and reduced trust in health professionals (20–22). These issues underscore the need for a judicious and selective integration of conversational LLMs in the healthcare setting (23).

## Data Availability

The original contributions presented in the study are included in the article/Supplementary material, further inquiries can be directed to the corresponding author.
