# Corrigendum: Neurological history both twinned and queried by generative artificial intelligence

**DOI:** 10.3389/fmed.2025.1619686

**Published:** 2025-05-27

**Authors:** Jung-Hyun Lee, Eunhee Choi, Sergio L. Angulo, Robert A. McDougal, William W. Lytton

**Affiliations:** ^1^Department of Neurology, State University of New York Downstate Health Sciences University, Brooklyn, NY, United States; ^2^Department of Neurology, Kings County Hospital, Brooklyn, NY, United States; ^3^Department of Neurology, Maimonides Medical Center, Brooklyn, NY, United States; ^4^Department of Internal Medicine, Lincoln Medical Center, Bronx, NY, United States; ^5^Department of Biostatistics, Yale School of Public Health, Yale University, New Haven, CT, United States; ^6^Computational Biology and Bioinformatics, Yale University, New Haven, CT, United States; ^7^Wu Tsai Institute, Yale University, New Haven, CT, United States; ^8^Biomedical Informatics and Data Science, Yale School of Medicine, Yale University, New Haven, CT, United States; ^9^Department of Physiology and Pharmacology, State University of New York Downstate Health Sciences University, Brooklyn, NY, United States

**Keywords:** neurology–clinical, stroke, headache, neurodegenerative disease, large language model (LLM), history taking

In the published article, the Acknowledgment was mistakenly not included in the publication. The missing Acknowledgment appears below:

