# Supplementary material for: Neurological history both twinned and queried by generative artificial intelligence
Source: Front Med (Lausanne). 2025 Jan 17;11:1496866. doi: 10.3389/fmed.2024.1496866 (PMC11782252; doi:10.3389/fmed.2024.1496866)
Supplement: Supplementary file 1 [file Presentation_1.pptx]

## Slide 1
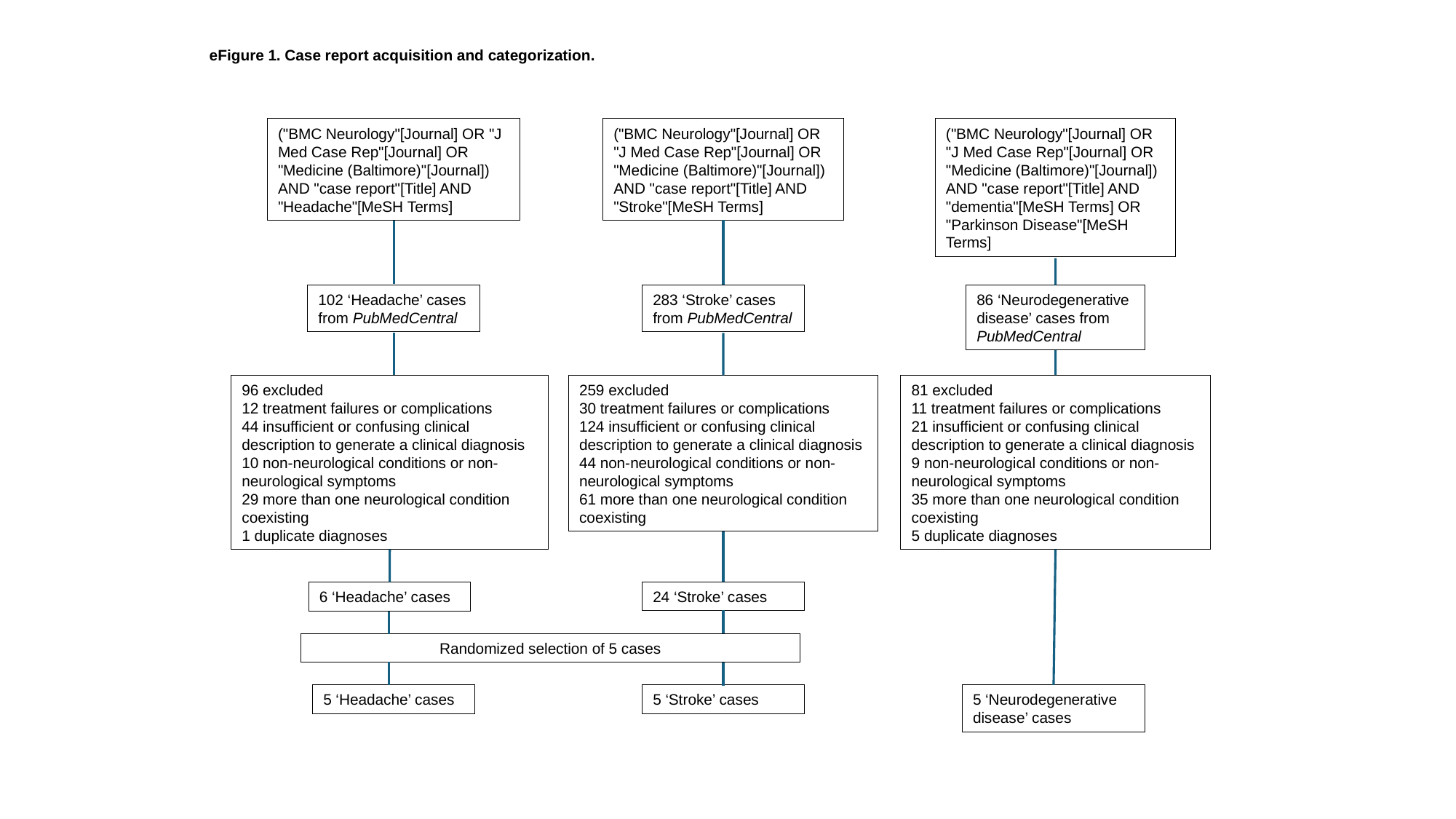

eFigure 1. Case report acquisition and categorization.
("BMC Neurology"[Journal] OR "J Med Case Rep"[Journal] OR "Medicine (Baltimore)"[Journal]) AND "case report"[Title] AND "Headache"[MeSH Terms]
("BMC Neurology"[Journal] OR "J Med Case Rep"[Journal] OR "Medicine (Baltimore)"[Journal]) AND "case report"[Title] AND "Stroke"[MeSH Terms]
("BMC Neurology"[Journal] OR "J Med Case Rep"[Journal] OR "Medicine (Baltimore)"[Journal]) AND "case report"[Title] AND "dementia"[MeSH Terms] OR "Parkinson Disease"[MeSH Terms]
102 ‘Headache’ cases from PubMedCentral
283 ‘Stroke’ cases from PubMedCentral
86 ‘Neurodegenerative disease’ cases from PubMedCentral
96 excluded
12 treatment failures or complications
44 insufficient or confusing clinical description to generate a clinical diagnosis
10 non-neurological conditions or non-neurological symptoms
29 more than one neurological condition coexisting
1 duplicate diagnoses
81 excluded
11 treatment failures or complications
21 insufficient or confusing clinical description to generate a clinical diagnosis
9 non-neurological conditions or non-neurological symptoms
35 more than one neurological condition coexisting
5 duplicate diagnoses
259 excluded
30 treatment failures or complications
124 insufficient or confusing clinical description to generate a clinical diagnosis
44 non-neurological conditions or non-neurological symptoms
61 more than one neurological condition coexisting
24 ‘Stroke’ cases
6 ‘Headache’ cases
Randomized selection of 5 cases
5 ‘Headache’ cases
5 ‘Stroke’ cases
5 ‘Neurodegenerative disease’ cases
